# Seasonal diversity of Cerambycidae (Coleoptera) is more complex than thought: evidence from a tropical dry forest of Mexico

**DOI:** 10.7717/peerj.7866

**Published:** 2019-10-18

**Authors:** José Guadalupe Martínez-Hernández, Angélica María Corona-López, Alejandro Flores-Palacios, Matthias Rös, Víctor Hugo Toledo-Hernández

**Affiliations:** 1 Centro de Investigación en Biodiversidad y Conservación, Universidad Autónoma del Estado de Morelos, Cuernavaca, Morelos, Mexico; 2 CONACyT, Centro Interdisciplinario de Investigación para el desarrollo Integral Regional, Instituto Politecnico Nacional, Oaxaca, Oaxaca, Mexico

**Keywords:** Seasonal distribution, Saproxilophagous, Dry tropics, True diversities

## Abstract

Global climate change is expected to affect temperature and precipitation patterns worldwide, which in turn is likely to affect insect phenology, distribution and diversity. To improve our understanding of such processes, it is important to understand how insects may respond to changes in seasonality, and how these affect their activity, patterns of distribution and species richness. The tropical dry forest (TDF) is a highly seasonal ecosystem, for which two seasons are commonly described (rainy and dry) and there is a lack of information on the combined effect of both precipitation and temperature on the insect communities. In order to evaluate the seasonal patterns in the community of Cerambycidae in a TDF, historical climatic variables were obtained, and an annual sampling of the family was carried out, using three collection techniques. We found that the Cerambycidae family showed a more complex response to climate, than simply the rainy and dry season of the year. The relationship between diversity and composition of cerambycids with changes in temperature and precipitation showed four seasonal communities which were synchronized with phenological processes of the TDF. Climate change could reduce biodiversity, causing seasonal patterns to lose complexity, either because the climatic characteristics of a season disappear and/or because the duration of a season expands, these changes will modify the ecological processes of the TDF, since they would generate changes in the flora and fauna associated with the different seasons.

## Introduction

Phenology is defined as the study of the timing of recurrent biological activities in order to detect their seasonal patterns and their causes (Rathcke & Lacey, 1985). Phenological patterns are critical for the survival and reproduction of species (Mantovani et al., 2003), due to their influence on ecosystem processes, particularly nutrient absorption, recycling and primary productivity, which are factors that promote the coexistence of species (Cleland et al., 2007).

The phenological variation of plant behavior is mainly related to weather factors and generates pulses of resources for animals, creating a seasonal segregation of animal species (Rathcke & Lacey, 1985; Cardoso et al., 2007). Insects are a suitable group for the study of seasonal patterns of diversity, since they show rapid responses to environmental changes and close relationships with vegetation phenology (Danks, 2007). However, for many insects groups and guilds, seasonal patterns of species diversity have been less studied than spatial patterns, and few published works show data of at least one complete annual cycle (comprising all the seasons Dornelas et al., 2013).

Insect phenology has been studied in different ecosystems, including environments with little climatic seasonality such as tropical humid forests, and there are more studies in environments that present a marked differentiation between the rainy and dry periods (Wolda, 1988; Zaragoza-Caballero et al., 2010; Novais et al., 2016). The seasonal pattern of insects depends on both the taxonomic group and the environment it inhabits (Kishimoto-Yamada & Itioka, 2015). The seasonal patterns are the result of natural selection, and it is considered that a seasonal pattern was selected to optimize survival, reproduction, or both, in the individuals of the populations, the current activity patterns are probably the result of phylogenetic inertia and reflect adaptations to ancestral conditions (Danks, 2007).

Tropical dry forest (TDF) is a seasonal environment, in which most rainfall occurs over a period of three to six months, in short and intense precipitation events. The decrease in the amount of rain during the dry season causes changes in the physiognomy of the forest, a reduction in productivity and in the availability of resources for animals, as a consequence, there is a corresponding decrease in the biological activity in the forests (Janzen, 1976; Lister & García, 1992; Pérez-García, Meave & Cevallos-Ferriz, 2012).

The TDF is considered the tropical forest most threatened by climate change. This high potential risk is caused by an estimated reduction of precipitation and a higher and more homogeneous temperature in the TDF areas of distribution (Miles et al., 2006). Changes in this ecosystem due to climatic stress will potentially alter their distribution and their phenological patterns and, consequently, the distribution and seasonality of the animal species that inhabit it (Mora et al., 2013; Macedo-Reis et al., 2016; Wright et al., 2017; Novais et al., 2018a).

In general, seasonality in the TDF is related to precipitation, since plant growth occurs mainly during the rainy season (Murphy & Lugo, 1986), and thus, the abundance and number of insect species increases during this period (Wolda, 1978; Pescador-Rubio, Rodríguez-Palafox & Noguera, 2002). However, other vegetation processes, such as flowering, fructification and seed dispersal occur in different periods during the year (Rathcke & Lacey, 1985; Murphy & Lugo, 1995; Pezzini et al., 2014).

In Mexico, studies on TDF have shown seasonal changes in the composition and structure of different insect communities (De la Maza, 2010; Zaragoza-Caballero et al., 2010). For example, in the case of the TDF located in the south of the state Morelos (central Mexico), studies show that the composition, abundance and species richness of several insect communities change between the dry and rainy seasons (Noguera et al., 2002; Zaragoza-Caballero, 2003; Rodríguez & Woolley, 2005; Ávalos, 2007; Corona-López et al., 2013; Toledo-Hernández et al., 2015; Corona-López et al., 2017).

Cerambycidae beetles are of great ecological importance in forest ecosystems. Their larvae are saproxilophagous (saprophagous/xylophagous), and thus they are key species in the process of wood decomposition (Noguera & Chemsak, 1996; Hovore, 2006; Calderón-Cortés, Quesada & Escalera-Vázquez, 2011; Novais et al., 2018b). The imagos (adult stage) of different species feed on sap, leaves, flowers, fruits, bark and fungi (Monné & Bezark, 2015). Due to their saproxilophagous behavior, it has been suggested that some species of this family have specific relationships with plant taxa, and thus, the moment of emergence of the imagos could be determined by the phenology of the host plants (e.g., optimizing the availability of oviposition sites, Vargas-Cardoso et al., 2018). In this way, the seasonal patterns of the emergence of Cerambycidae imagos could reflect an optimal time for their reproduction (Keszthelyi, 2015; Monné & Bezark, 2015). Despite this general pattern, there have been very few studies about the annual patterns of imago activity of the Cerambycidae, especially in TDF.

Knowing the temporal changes in diversity, as the time of species occurrence, duration, synchrony, and predictability of each phenophase and their relationship with climatic variables are key pieces of information for biodiversity management and conservation. Climate change caused by anthropogenic activities will affect the climatic variables that signal the onset of phenophases, affecting the temporary replacement of species (Moreno & Halffter, 2001; Lee et al., 2014).

We survey the diversity and seasonal patterns of the community of Cerambycidae in a TDF of the central southern region of Mexico. We expected to find concordance between the seasonal dichotomy (rainy and dry periods) of the TDF, and the behavior of the Cerambycidae community. We hypothesized that the composition and diversity of the Cerambycidae community would reflect two seasonal communities, based on the emergence of their imagos, and that this behavior would be related with precipitation and temperature.

## Materials & Methods

### Study site

**Figure 1:**
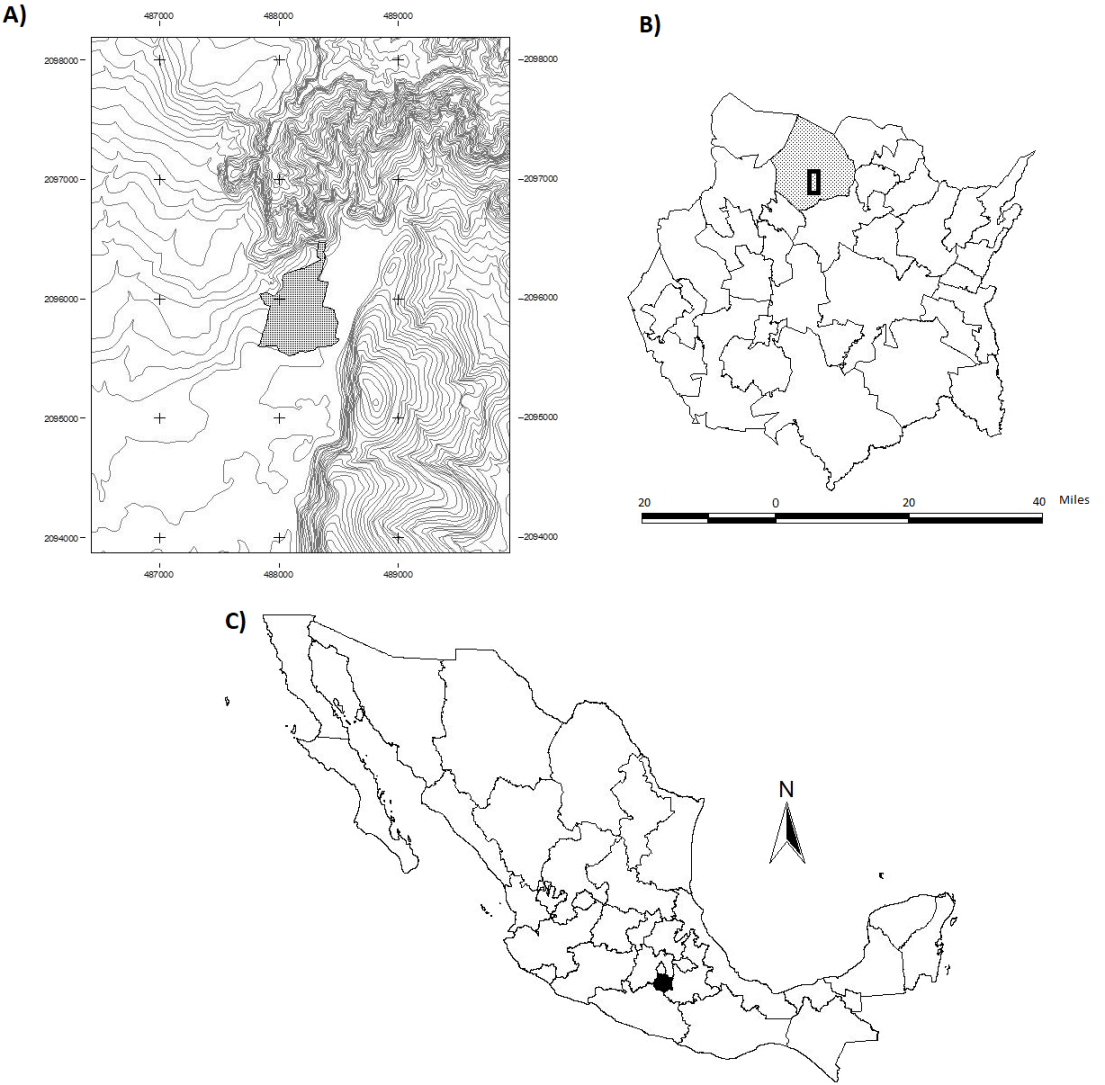
Geographical location of the study area. The geographical location of the study area (UTM): (A) San Andrés de la Cal (contours and the polygon is the urban area), (B) municipality of Tepoztlán in the state of Morelos (C) Mexico.

This work was conducted in the TDF of the ejido of San Andrés de la Cal, Tepoztlán, Morelos, México (18°57′22.2″N, 99°06′50.2″W; Fig. 1). This TDF is protected and forms part of the “El Tepozteco” National Park (CONANP, 2008; INEGI, 2015). The area presents a rugged topography with an altitudinal gradient of 1,300–1,770 m a.s.l. The climate is semi-warm, with an annual mean temperature of 20 °C, and a mean annual precipitation of 1,200 mm, with rains from May to October. The TDF in the zone is formed by at least 85 species of woody plants in two forest units; one developed on limestone rock, in which the dominant species are *Sapium macrocarpum* Müll. Arg. (Euphorbiaceae), *Bursera fagaroides* (Kunth) Engl., *B. glabrifolia* (Kunth) Engl. (Burseraceae) and *Conzattia multiflora* (B. L. Rob.) Standl. (Fabaceae), and the other on volcanic rock, in which the dominant species are *S. macrocarpum*, *Ipomoea pauciflora* M. Martens and Galeotti (Convolvulaceae) and *Quercus obtusata* Bonpl. (Fagaceae) (Vergara-Torres, Pacheco-Álvarez & Flores-Palacios, 2010; Cortes-Anzures et al., 2017).

### Collection methods

Diurnal and nocturnal sampling was conducted monthly over six consecutive days from February 2015 to January 2016 (SEMARNAT collection permit: FAUT-0178), covering the rainy (May, June, July, August, September and October) and dry (November, December, January, February, March and April) seasons. The diurnal collections (10:00–15:00 h) were done by four persons, who made *ad libitum* trips searching for cerambycid individuals on flowers, branches and fallen trunks, as well as beating the vegetation (trees, shrubs and herbs) with an entomological net. Total diurnal sampling effort was 1,440 h (4 persons × 5 h × 6 days × 12 months).

In the nocturnal collections (4 h per night, and the periods varied according to the setting of the sun), a screen light trap (2 m^2^ wide, two 175 watt mercury vapor lamps), was used. To increase sample representation, the light trap was set in a different site every night, but the same sites were repeated every month. Total nocturnal sampling effort was 288 h (1 trap ×4 h ×6 nights ×12 months).

Collected specimens were dry-conserved with entomological pins and deposited in the Insect Collection of the Autonomous University of the State of Morelos (CIUM), located in the Centro de Investigación en Biodiversidad y Conservación (CIβγC) of the Autonomous University of the State of Morelos (UAEM).

In order to understand the climatic variability of the study area, climatic data (precipitation and temperature) pertaining to the zone was compiled. This information was obtained from the nearest climatological station of the Mexican National Water Commission (station number 17049, CONAGUA, unpublished data).

### Data analyses

Sampling efficiency was evaluated using sample coverage ($\hat {C}m$); sample coverage measures the completeness of an inventory in terms of the species abundances observed, and enables interpolation/extrapolation of the richness expected, based on the estimator Chao 1, allowing the comparison of richness between communities at the same coverage (Chao & Jost, 2012).

The diversities for the entire sampling, for each sampling unit (i.e., month) and for each season, were calculated using ^*q*^D true diversities (Jost, 2006; Jost, 2007). These diversities were calculated for the alpha and beta levels at three different orders of *q* (0, 1, 2), with *q* indicating the sensitivity of the measure of diversity to the abundance of the species. When *q* is equal to 0, ^0^D ignores species abundance and its values are equal to species richness. When *q* is equal to 1, ^1^D perfectly weights the species importance according to their proportional abundances, and ^1^D is equal to the exponential of the Shannon diversity index. When *q* is equal to 2, ^2^D overweights the species proportional abundance, and ^2^D is equivalent to the inverse of the Simpson dominance index (Jost, 2006). To measure the evenness, the proportion of rare species was calculated (1-EF _0,2_) as well as the relative logarithmic inequality (RLI _0,2_, Jost, 2010). The true diversities and sample coverages were calculated using the package Entropart (Marcon & Hérault, 2015) in the statistical program R (version 3.3.2).

In order to determine whether seasonality generated compositional changes along the year in the community of cerambycids, two analyses were performed with the sampling months. The first was non-metric multidimensional scaling analysis (NMDS; Dunn & Everitt, 2004) and the second was an analysis of beta diversity (Jost, 2007). For the NMDS analysis a matrix was constructed with the paired values of the Morisita-Horn similarity index among the sampling months (supplementary material). This similarity index is directly related to the values of ^2^D diversity, and accordingly was calculated using the equation for compositional similarity among communities (Jost, 2007; Jost, Chao & Chazdon, 2011). In order to interpret the NMDS ordering of the sampling units (months), clustering patterns were graphically sought, and the NMDS dimensions were correlated (Pearson) with the abundance, diversity and variables of average monthly precipitation and temperature.

For the beta diversity analysis, we first calculated the gamma (^*q*^*D*_γ_) and alfa (^q^*D*_α_) diversities and estimated beta as ^*q*^*D*_γ_∕^*q*^*D*_α_, following Jost (2007). For this analysis we used the same values of q and the R-package referred before (Entropart, Marcon & Hérault, 2015).

## Results

A total of 1,570 individuals of 126 species were collected, belonging to four subfamilies, 35 tribes and 81 genera ( Complementary Material). The coverage for the entire sampling was 97%, which suggests that the 34 non-collected species (according to the estimator Chao 1) only represented 3% of the individuals of the community. Sampling coverage for each month varied from 75–98% for June and October, respectively (Table 1).

**Table 1:** Values by seasonal subgroups. Values of diversities, abundance, sample coverage of Cerambycidae, the environmental variables temperature and precipitation (sd = standard deviation) and months per seasonal subgroup in San Andrés de la Cal.

**Seasonal subgroup**	**Month**	^0^*D*	^1^*D*	^2^*D*	**Abundance**	Cm	**Temperature (±sd)**	Precipitation (±sd)
Late dry	Feb	12	6.8	5.1	80	95.06	22 ± 2.3	61.5 ± 4.9
	Mar	13	8.1	5.9	42	83.56	23 ±.5 2.2	46.5 ± 2
	Apr	15	11.1	8.3	38	84.89	23.5 ± 2.3	98.5 ± 3.4
Early rainy	May	31	19.7	12.7	86	80.34	21.8 ± 2.7	187.1 ± 5.5
	Jun	61	44.7	31.2	129	75.40	20.5 ± 2.4	528.8 ± 14.6
Late rainy	Jul	39	22.4	13.7	219	95.46	20.5 ± 1.8	480.5 ± 13.6
	Aug	35	13.3	7.0	182	90.13	20 ± 1.6	524.1 ± 14.5
	Sep	32	8.5	3.8	245	94.71	20 ± 1.7	513.1 ± 15
	Oct	27	14.9	9.0	211	98.60	19.7 ± 1.7	250.7 ± 9.8
	Nov	23	8.5	4.4	122	90.20	19.2 ± 2.3	68.5 ± 3.2
Early dry	Dec	13	4.9	2.9	126	96.85	19.6 ± 1.8	67.9 ± 1.3
	Jan	6	1.5	1.2	90	95.59	20.5 ± 2.1	63.3 ± 3.4

**Figure 2:**
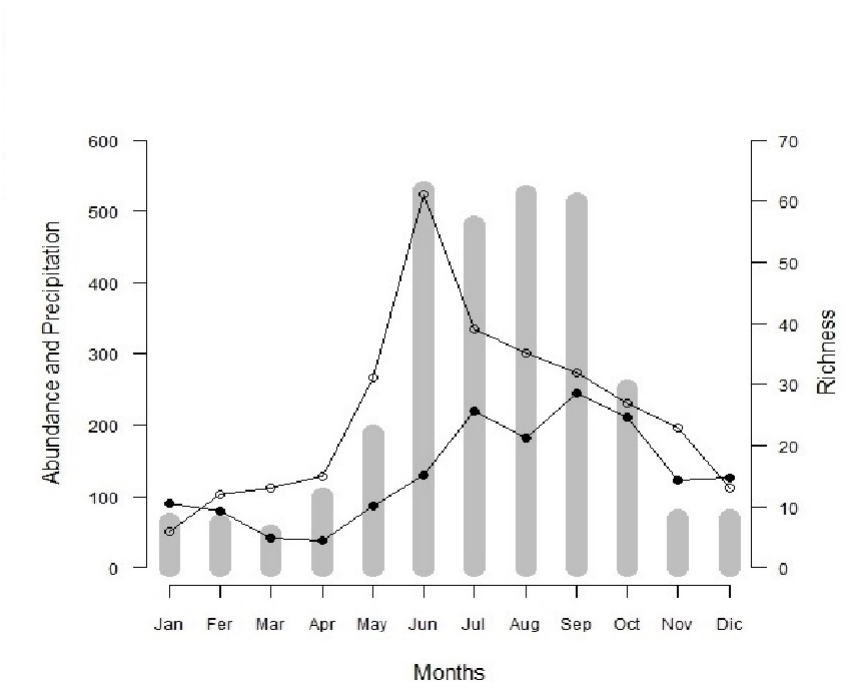
Richness, abundance and precipitation of San Andrés de la Cal per month. Monthly Richness (white circles) and abundance (black circles) of the Cerambycidae community of San Andrés de la Cal, Tepoztlán, Morelos. The bars are the historical average (25 years) of monthly precipitation (mm).

The activity of the species of Cerambycidae displayed a marked seasonality; greatest richness (June, 61 spp) and abundance (September, 245 individuals), were found during the rainy season (Fig. 2, Table 1). A total of 66% of the species were active during the rainy season (89 spp), 20% were recorded in both seasons (26 spp) and 14% were only present in the dry season (19 spp). A total of 84% of the species were only present between one and three months.

The NMDS analysis required two dimensions to arrange the samples (stress = 0.089). Dimension 1 correlated negatively with temperature (*r* =  − 0.78, *p* = 0.002), and dimension 2 positively with precipitation (*r* = 0.79, *p* = 0.005), with species richness (*r* = 0.67, *p* = 0.028) and abundance (*r* = 0.87, *p* = 0.001) of the cerambycids.

**Figure 3:**
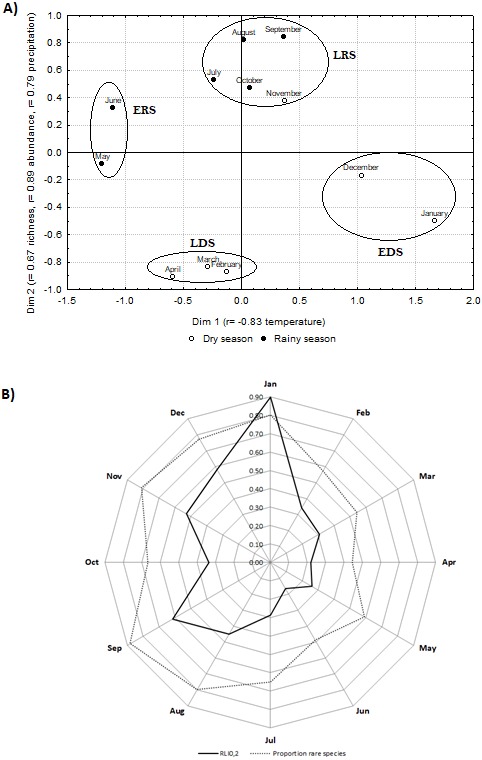
Non-metric multidimensional scale, relative logarithmic inequality values and the proportion of rare species. Non-metric multidimensional scaling (NMDS) ordering of the community of cerambycids in a TDF of central Mexico (black dots: months of the rainy season and white dots: months of the dry season). (A) The ordering is based on a matrix of similarity among pairs of months using the Morisita-Horn index (stress 0.089), the circles represent the seasonal subgroups, ERS (early rainy season), LRS (late rainy season), EDS (early dry season) and LDS (late dry season). (B) The monthly values of the relative logarithmic inequality (RLI_0,2_, continuous line) and the proportion of rare species (1-EF_0,2_, dotted line) are shown.

Four seasonal groups were observed in the NMDS ordering (Fig. 3A). These four seasonal groups are: (1) the early rainy season (ERS- May and June), (2) the late rainy season (LRS- July, August, September, October and November), (3) the early dry season (EDS- December and January), and (4) the late dry season (LDS- February, March and April). Comparison of the similarity values between pairs of months showed higher similarity among the months of each seasonal group (Complementary material), than between groups; except for the group formed by the months of May and June, which presented the lowest values in comparison with the other seasonal groups.

The presence of four seasonal communities were also supported by the beta diversity analysis, since all the beta diversities values (^0^D_*β*_ = 4.32, ^1^D_*β*_ = 3.63 and ^2^D_*β*_ = 3.79) round to 4, indicating the existence of four seasonal communities in the study area.

Each of the four seasonal communities suggested by both analyses (NMDS and ^*q*^*D*_β_) can be described by its own characteristics of diversity of Cerambycidae and climatic variables (Fig. 3B; Table 1). The ERS occurred at the beginning of the rainy season, with a mean temperature of 22 °C, and in this season the values of diversity were the highest and inequality was the lowest. The LRS occurred during the peak of precipitation, with a mean temperature of 20 °C, and the greatest proportion of individuals and rare species were detected. The EDS occurred when precipitation was less than 10 mm and the mean temperature is 19 °C, the diversity was the lowest and inequality was the highest. Finally, the LDS occurred when precipitation did not exceed 40 mm, mean temperature is 21 °C, in the LDS emerged both the lowest proportion of individuals and of rare species (Fig. 3B; Table 1).

## Discussion

In the TDF, the strongly seasonal climate has led to the general recognition of two seasons (rainy and dry seasons), in which the plants concentrate different periods of their growth, (Murphy & Lugo, 1986). This simplification does not take into account that both plants and animals could respond to climatic variations within these defined seasons. Our data indicate that both the climate of the study zone and the behavior of the Cerambycidae community are divided into four seasons.

Despite the highest sampling coverage obtained, the estimate of species richness suggests that 20% of species in the study area were not collected. This is consistent with other studies, where the expected number of cerambycid species not recorded was between 27–50%, according to ICE and Chao 2 (Noguera et al., 2002; Noguera et al., 2007; Noguera et al., 2009; Noguera et al., 2012; Toledo et al., 2002).

The ^*q*^*D* diversity values are lower than those obtained in three nearby southern TDF sites (Coaxitlán, Huaxtla and El Limón de Cuauchichinola) of the Sierra de Huautla biosphere reserve (REBIOSH); where the species richness recorded were 204, 140 and 141 species of cerambycids, respectively (Martínez-Hernández, 2013). This may indicate that the diversity of cerambycids is lower in the TDF of north of the state of Morelos, possibly because of the more temperate climate in comparison with that of the south and is in accordance with the fact that species of cerambycids prefer average temperatures around 23 °C (Danks, 2007; Lee et al., 2014).

The climatic seasonality of the TDF has normally been characterized in two seasons, and the general phenological patterns of this vegetation type are thus described (Murphy & Lugo, 1986; Murphy & Lugo, 1995). From this pattern, the TDF has been understood as a simple and predictable ecosystem, and it is also assumed that the communities of insects relate only to these two seasons (Rodríguez & Woolley, 2005; Ávalos, 2007; Corona-López et al., 2013). However, the results of this study show that the climatic variability in the TDF is more complex, and that four different communities of cerambycids correspond to it.

Comparing the seasonal communities, we found that the greatest values of ^q^*D*_β_ diversity were obtained for ^0^*D*_β_. This indicates that the greatest differentiation between seasons is due to the species that are found in each season, that is, the seasons show differences in species composition, rather than differences in abundance or species dominance between the seasons. The multidimensional scaling analysis reaffirms this seasonal pattern, since the samples display a configuration that correlates with both precipitation and temperature; and the relationship with precipitation and temperature generates four seasonal groups.

Other cerambycid communities also show more than two seasonal communities. In the conifer forests of northeastern USA, the cerambycid community presented a seasonal pattern divided into three groups, which are related to the seasons of spring (March, April and May), summer (June, July and August) and autumn (September, October and November), the activity in winter was not sampled due to the characteristic snowfall of the area (Hanks et al., 2014). These three seasonal periods found in the conifer forests are neither attributed to environmental variables nor to the phenology of vegetation (Hanks & Millar, 2013; Handley et al., 2015). For this vegetation type in North America, temperature is recognized as the main driver of seasonal patterns in the poikilothermic animals, and in insects, this is achieved through regulation of the duration of the diapause (Powell et al., 2000).

A dichotomic seasonal (transition between rainy and dry seasons) pattern differentiated by precipitation, has been observed in tropical regions for various groups of insects (Kishimoto-Yamada & Itioka, 2015; Novais et al., 2016) and for different families of beetles in the TDF (Toledo-Hernández et al., 2015; Macedo-Reis et al., 2016; Corona-López et al., 2017; Novais et al., 2018b). For the Cerambycidae communities, this pattern has been recognized in the TDF of different regions of Mexico (Noguera et al., 2002; Noguera et al., 2007; Noguera et al., 2009; Noguera et al., 2012; Toledo et al., 2002). The relationship between the activity of the insects of the TDF and precipitation and temperature can be explained by the fact that availability of water limits primary productivity, while both limit the decomposition rate of wood (Maass et al., 2002; Jaramillo, Martínez-Yrízar & Sanford-Jr, 2011), and temperature affects the metabolism of the insects (Danks, 2007; Müller et al., 2015).

The observed seasonal subgroups of cerambycids can also be related with phenological processes of the vegetation. The ERS cerambycids could be related with the foliation of woody plants, the LRS with the flowering of herbaceous plants (which occurs mainly from September to November (Corona-López et al., 2017), the EDS with the flowering of woody plants, and the LDS with the fructification of woody plants (Rathcke & Lacey, 1985). A similar pattern occurs in the TDF of Chamela, Jalisco, where these subgroups coincide with foliation (LRS), flowering (ERS), fructification (EDS) and seed dispersion (LDS, Bullock & Solis-Magallanes, 1990; Wright et al., 2017). However, it is also possible that the presence of four seasonal subgroups of cerambycids could be due to phylogenetic inertia, resembling the phenological adaptation to past climate and suggesting that this cerambycid community lacks of adaptative capacity to the present climate.

Changes in the seasonality (phenology) of TDF due to changes in weather patterns, will generate in insect communities changes in their activity patterns, migrations to suitable microhabitats and even local extinctions, since changes in humidity affect the duration of larval development and adult fecundity (Jaworski & Hilszczanski, 2013).

While the increase in temperature causes greater drying and an increase in metabolism rate, these changes in activity or the disappearance of insect species could cause changes in the higher trophic levels and favor the emergence of opportunistic or invasive species (Chown, Sørensen & Terblanche, 2011). In the particular case of saproxilophagousity, due to its high specificity of habitat, the effects of changes in climatic patterns are expected to be more strong, affecting ecosystem services, particularly the recycling of nutrients due to the degradation of dead wood (Berkov, 2018).

Global temperatures are expected to increase by 1–3 °C during the next 100 years (IPCC, 2013), and may increase the length of the dry season. That could favor the late-dry community of Cerambycidae, because it is associated with the highest temperature and the lowest precipitation, but this community is the less diverse, while the early dry community appeared when temperature was 2–3 °C lower. The community of Cerambycidae that appeared during the rainy season also occurred when temperatures were 2–3  °C lower than the late-dry community, suggesting that climate change could affect the most diverse communities of Cerambycidae of the TDF.

## Conclusions

The results of this study confirm the relationship between climate seasonality and the emergence periods of cerambycids, and how these seasonal patterns are recognized, both by precipitation and by temperature. The TDF is an ecosystem of wide distribution but is considered as one of the most threatened by deforestation, fragmentation and climatic change (Miles et al., 2006; Kegan, 2014). Efforts such as this work, can help to increase our knowledge in terms of this complex ecosystem, and thus, contribute to its conservation. Further research that can contribute to elucidating the ecological (pulses of resources) or evolutionary (phylogenetics) causes behind these four seasons is necessary, in order to fully understand the causes of this behavior in the cerambycids and predict the response of these group of insects to climate change.

##  Supplemental Information

10.7717/peerj.7866/supp-1 Supplemental Information 1 Abundance, precipitation and temperature data per month of the morphospecies of Cerambycidae in San Andres de la Cal, Tepoztlán, Morelos, Mexico

10.7717/peerj.7866/supp-2 Supplemental Information 2 Species List

10.7717/peerj.7866/supp-3 Table S1 Similarity matrix used for NMDS Morisita-Horn matrix of similarity of Cerambycidae recorded in study area.

## Additional Information and Declarations

### Field Study Permissions

The following information was supplied relating to field study approvals (i.e., approving body and any reference numbers): Secretaria de Medio Ambiente y Recursos Naturales, SEMARNAT gave the permission for insect collection (FAUT-0178).

### Data Availability

The following information was supplied regarding data availability: Raw data can be found in the Supplemental File.
