# Supplementary material for: Seasonal diversity of Cerambycidae (Coleoptera) is more complex than thought: evidence from a tropical dry forest of Mexico
Source: PeerJ. 2019 Oct 18;7:e7866. doi: 10.7717/peerj.7866 (PMC6802581; doi:10.7717/peerj.7866)
Supplement: Supplemental Information 2 [file peerj-07-7866-s002.docx]

**Species list**

Cerambycidae species recorded in San Andrés de la Cal, Tepoztlán, Morelos, Mexico, according to the taxonomic classification of Monné (2019a); Monné (2019b); Monné (2019c) and Tavakilian (2019), including the collection season: early rainy season (ERS ), late rainy season (LRS), early dry season (EDS) and late dry season (EDS).

Prioninae

Mallodonini

*Nothopleurus lobigenis* Bates, 1884, ERS, LRS and LDS.

Lepturinae

Lepturini

Strangalia doyeni Chemsak & Linsley, 1976, LRS.

Cerambycinae

Callochromatini

*Plinthocoelium chilensis* (Blanchard, 1851), ERS.

Cerambycini

*Jupoata rufipennis* (Gory, 1831), ERS

Clytini

*Neoclytus cacicus* (Chevrolat, 1860), ERS and LDS

*Placosternus difficilis* (Chevrolat, 1862), ERS and EDS.

*Tanyochraethes truquii* (Chevrolat, 1860), ERS.

Dryobiini

*Ornithia zapotensis* Tippmann, 1960, LRS.

Eburiini

*Eburodacrys nemorivaga* Gounelle, 1909, LRS.

*Susuacanga poricollis* (Chemsak & Linsley, 1973), ERS.

Elaphidiini

*Aneflomorpha rectilinea* Casey, 1924, ERS.

*Aneflomorpha* sp.1, LRS.

*Anelaphus badius* Chemsak, 1991, LDS.

*Anelaphus hirtus* Chemsak & Noguera, 2003, LRS.

*Anelaphus nitidipennis* Chemsak & Linsley, 1968, LDS.

*Anelaphus piceus* (Chemsak, 1962), LDS.

*Anelaphus vernus* Chemsak, 1991, LDS.

*Conosphaeron concolor concolor* Linsley, 1935, LRS.

*Metironeus hesperus* Chemsak, 1991, LRS.

*Micropsyrassa pilosella* (Bates, 1892), LRS.

*Psyrassa megalops* Chemsak & Noguera, 1993, LRS.

*Stenosphenus cribripennis cribripennis* Thomson, 1861, ERS and LRS.

*Stenosphenus sobrius* (Newman, 1840), LRS.

*Stenosphenus trispinosus* Bates, 1872, ERS and LRS.

*Trichophoroides* sp.1, LRS.

Hesperophanini

*Makromastax mandibularis* (Chemsak & Linsley, 1963), LDS.

*Xeranoplium ruficolle* Chemsak & Linsley, 1963, LRS.

Hexoplonini

*Hexoplon calligrammum* Bates, 1885, ERS and LRS.

Lissonotini

*Lissonotus flavocinctus* Dupont, 1836, LRS.

Methiini

*Methia bicolorata* Linsley, 1962, LRS.

*Methia lineata* Linsley, 1935, LDS and EDS.

*Styloxus angelesae* Noguera, 2005, LRS and LDS.

Neoibidionini

*Neocompsa agnosta* Martins, 1970, LRS.

*Neocompsa alacris* (Bates, 1885), ERS.

*Neocompsa clerochroa* (Thomson, 1867), ERS and LRS.

*Neocompsa puncticollis asperula* (Bates, 1885), ERS and LRS.

Obriini

*Obrium cruciferum* Bates, 1885, ERS.

Rhinotragini

*Ameriphoderes cribricollis* (Bates, 1873), LRS.

*Odontocera* sp.1, LRS.

*Rhinobatesia rugicollis* (Bates, 1880), LRS.

Rhopalophorini

*Cycnoderus* (*Cycnoderus*) *lividus*, Giesbert & Chemsak, 1993, ERS.

*Rhopalophora serripennis* Giesbert & Chemsak, 1993, LRS.

*Rhopalophora tenuis* (Chevrolat, 1855), ERS and LRS.

Tillomorphini

*Euderces cribripennis* Bates, 1892, LRS.

*Euderces pulcher* (Bates, 1874), LRS.

Trachyderini

*Ancylocera michelbacheri* Chemsak, 1963, LRS.

*Assycuera macrotela* (Bates, 1880), ERS and LRS.

*Callistochroma rutilans* (Bates, 1869), ERS.

*Deltaspis rubriventris* Bates, 1880, ERS.

*Dendrobias mandibularis mandibularis* Dupont, 1834, LRS.

*Elytroleptus scabricollis* Bates, 1892, LRS.

*Ischnocnemis caerulescens* Bates, 1885, ERS.

*Ischnocnemis sexualis* Bates, 1885, ERS.

*Ischnocnemis virescens* Eya, 2010, ERS.

*Muscidora tricolor* Thomson, 1864, ERS.

*Sphaenothecus picticornis* Bates, 1880, EDS.

*Sphaenothecus trilineatus* Dupont, 1838, ERS and EDS.

*Stenaspis verticalis verticalis* Audinet-Serville, 1834, ERS.

*Stenobatyle eburata* (Chevrolat, 1862), ERS and LRS.

*Trachyderes elegans elegans* Dupont, 1836, ERS and LRS.

*Tylosis puncticollis* Bates, 1885, ERS, LRS and .EDS

Lamiinae

Acanthocinini

*Canidia canescens* (Dillon, 1955), ERS.

*Canidia mexicana* Thomson, 1861, LRS.

*Canidia spinicornis* (Bates, 1881), ERS.

*Eutrichillus brevipilus* Chemsak & Linsley, 1986, ERS and LRS.

*Eutrichillus comus* (Bates, 1881), ERS, LRS, LDS and EDS.

*Lagocheirus araneiformis ypsilon* (Voet, 1778), ERS, LRS and EDS.

*Lagocheirus obsoletus obsoletus* Thomson, 1778, ERS, LRS, LDS and EDS.

*Leptostylus dubitans* Bates, 1885, LDS.

*Lepturges* (*Lepturges*) *infilatus* Bates, 1872, LRS.

*Lepturges* sp.1, LRS.

*Mecotetartus antennatus* Bates, 1872, ERS, LRS and LDS.

*Olenosus serrimanus* Bates, 1872, ERS, LRS, LDS and EDS.

*Proxatrypanius rockefelleri* Gilmour, 1959, ERS.

*Trichalphus pilosus* Bates, 1881, LDS.

*Urgleptes celtis* (Schaeffer, 1905), LRS.

*Urgleptes* sp.2, LDS.

Acanthocinini sp.3, LRS.

Acanthocinini sp.4, LRS.

Acanthocinini sp.6, LRS.

Acanthocinini sp.8, ERS and LRS.

Acanthocinini sp.9, ERS.

Acanthocinini sp.11, LRS.

Acanthoderini

*Acanthoderes* (*Pardalisia*) *lacrymans* (Thomson, 1864), ERS and LRS.

*Aegomorphus albosignus* Chemsak & Noguera, 1993, ERS, LRS and LDS.

*Psapharochrus borrei* (Dugés, 1885), ERS, LRS, LDS and EDS.

*Psapharochrus* sp.1, LRS

*Psapharochrus* sp.2, ERS and LDS.

*Psapharochrus* sp.3, LRS, LDS and EDS.

*Tetrasarus pictulus* Bates, 1880, ERS.

Agapanthiini

*Spalacopsis* sp.1, LRS.

Anisocerini

*Thryallis sallaei* Bates, 1880, ERS.

Apomecynini

*Adetus pisciformis* (Thomson, 1868), ERS.

*Adetus modestus* Melzer, 1934, ERS

*Dorcasta dasycera* (Erichson, 1848), ERS, LRS and EDS.

*Ptericoptus intermedius* Breuning, 1939, ERS.

Calliini

*Paradrycothaea pilosicornis* Galileo & Martins, 2010, ERS, LRS, LDS and EDS.

Colobotheini

*Colobothea sinaloensis* Giesbert, 1979, ERS.

Desmiphorini

*Desmiphora* (*Desmiphora*) *hirticollis* (Olivier, 1795), LDS.

*Estoloides chamelae* Chemsak & Noguera, 1995, ERS and EDS.

Estoloides nayeliae Santos-Silva, Wappes & Galileo, 2018, ERS and LRS.

*Eupogonius* sp.2, LRS.

Hemilophini

*Alampyris fuliginea* Bates, 1881, ERS.

*Essostrutha binotata* Bates, 1881, ERS and LRS.

*Essostrutha laeta* (Newman, 1840), ERS and LRS.

Moneilemini

*Moneilema albopictum* White, 1856, ERS.

Monochamini

*Chyptodes dejeani* (Thomson, 1865), ERS, LRS and EDS.

*Mimolochus hoefneri* (Thomson, 1865), ERS.

Onciderini

*Cacostola janzeni* Chemsak & Linsley, 1986, LRS.

*Lochmaeocles cornuticeps federalis* Dillon & Dillon, 1946, ERS and LRS.

*Taricanus truquii* Thomson, 1868, ERS and EDS.

Phytoeciini

*Mecas* (*Mecas*) *cinerea* (Newman, 1840), ERS.

*Mecas* (*Mecas*) *obereoides* Bates, 1881, ERS.

Pogonocherini

*Poliaenus hesperus* Chemsak & Linsley, 1988, ERS.

Pteropliini

*Ataxia perplexa* (Gahan, 1892), ERS and LRS.

Tetraopini

*Phaea biplagiata* Chemsak, 1977, LRS.

*Phaea bryani* Chemsak, 1999, ERS.

*Phaea lateralis* Bates, 1881, ERS and LRS.

*Phaea laurieae* Chemsak, 1999, ERS.

*Phaea paralella* Toledo, Martínez & Bezark, 2016, ERS and LRS.

*Phaea rufiventris* Bates, 1872, ERS and LRS.

*Phaea tenuata* Bates, 1872, LRS.

*Phaea vitticollis* Bates, 1872, ERS and LRS.

*Tetraopes cleroides* Thomson, 1860, ERS.

*Tetraopes discoideus* LeConte, 1858, ERS and LRS.

*Tetraopes umbonatus* LeConte, 1852, ERS and LRS.

**References**

**Monné MA. (2019a).** Catalogue of the Cerambycidae (Coleoptera) of the Neotropical Region. Part I. Subfamily Cerambycinae. http://cerambyxcat.com/Parte1_Cerambycinae_2018.pdf/ Accessed 3 September 2019

**Monné MA. (2019b).** Catalogue of the Cerambycidae (Coleoptera) of the Neotropical Region. Part II. Subfamily Lamiinae. http://cerambyxcat.com/Parte2_Lamiinae_2018.pdf/ Accessed 3 September 2019

**Monné MA. (2019c).** Catalogue of the Cerambycidae (Coleoptera) of the Neotropical Region. Part III. Subfamilies Lepturinae, Necydalinae, Parandrinae, Prioninae, Spondylidinae and Families Oxypeltidae, Vesperidae and Disteniidae. Necydalinae Parandrinae Prioninae Spondylidinae Oxypeltidae Vesperidae and Disteniidae. http://cerambyxcat.com/Parte3_Prioninae_Lepturinae_2018.pdf/ Accessed 3 September 2019

**Tavakilian G. (2019).** Base de données Titan sur les Cerambycides ou Longicornes. http://titan.gbif.fr/ Accessed 3 September 2019
