# Supplementary material for: Seasonal diversity of Cerambycidae (Coleoptera) is more complex than thought: evidence from a tropical dry forest of Mexico
Source: PeerJ. 2019 Oct 18;7:e7866. doi: 10.7717/peerj.7866 (PMC6802581; doi:10.7717/peerj.7866)
Supplement: Table S1 — Morisita-Horn matrix of similarity of Cerambycidae recorded in study area. [file peerj-07-7866-s003.docx]

**S1 Table. Morisita-Horn matrix of similarity of Cerambycidae recorded in study area.**

|  | January | February | March | April | May | June | July | August | September | October | November | December |
| --- | --- | --- | --- | --- | --- | --- | --- | --- | --- | --- | --- | --- |
| January | 1 | 0.009 | 0.008 | 0.006 | 0 | 0.001 | 0.005 | 0.006 | 0.01 | 0.01 | 0.068 | 0.873 |
| February | 0.009 | 1 | 0.772 | 0.332 | 0.149 | 0.082 | 0.182 | 0.082 | 0.045 | 0.112 | 0.079 | 0.052 |
| March | 0.008 | 0.772 | 1 | 0.502 | 0.174 | 0.163 | 0.133 | 0.047 | 0.038 | 0.163 | 0.122 | 0.092 |
| April | 0.006 | 0.332 | 0.502 | 1 | 0.175 | 0.174 | 0.056 | 0.019 | 0.014 | 0.186 | 0.146 | 0.021 |
| May | 0 | 0.149 | 0.174 | 0.175 | 1 | 0.228 | 0.194 | 0.083 | 0.03 | 0.089 | 0.066 | 0.038 |
| June | 0.001 | 0.082 | 0.163 | 0.174 | 0.228 | 1 | 0.251 | 0.083 | 0.042 | 0.115 | 0.079 | 0.023 |
| July | 0.005 | 0.182 | 0.133 | 0.056 | 0.194 | 0.251 | 1 | 0.724 | 0.664 | 0.733 | 0.658 | 0.141 |
| August | 0.006 | 0.082 | 0.047 | 0.019 | 0.083 | 0.083 | 0.724 | 1 | 0.806 | 0.753 | 0.669 | 0.128 |
| September | 0.01 | 0.045 | 0.038 | 0.014 | 0.03 | 0.042 | 0.664 | 0.806 | 1 | 0.818 | 0.897 | 0.176 |
| October | 0.01 | 0.112 | 0.163 | 0.186 | 0.089 | 0.115 | 0.733 | 0.753 | 0.818 | 1 | 0.87 | 0.155 |
| November | 0.068 | 0.079 | 0.122 | 0.146 | 0.066 | 0.079 | 0.658 | 0.669 | 0.897 | 0.87 | 1 | 0.251 |
| December | 0.873 | 0.052 | 0.092 | 0.021 | 0.038 | 0.023 | 0.141 | 0.128 | 0.176 | 0.155 | 0.251 | 1 |
